# Molecular Characterization of Hovenia Dulcis-Associated Virus 1 (HDaV1) and 2 (HDaV2): New Tentative Species within the Order *Picornavirales*

**DOI:** 10.3390/v12090950

**Published:** 2020-08-27

**Authors:** Flávia M. B. Nery, Fernando L. Melo, Leonardo S. Boiteux, Simone G. Ribeiro, Renato O. Resende, Anelise F. Orílio, Josiane G. Batista, Mirtes F. Lima, Rita C. Pereira-Carvalho

**Affiliations:** 1 Departamento de Fitopatologia, Universidade de Brasília (UnB), Campus Universitário Darcy Ribeiro, Brasília DF 70910-900, Brazil; flamilene.nery@gmail.com (F.M.B.N.); 05josiane@gmail.com (J.G.B.); 2 Embrapa Hortaliças, Brasília DF 70275-970, Brazil; leonardo.boiteux@embrapa.br (L.S.B.); mirtes.lima@embrapa.br (M.F.L.); 3 Embrapa Recursos Genéticos e Biotecnologia, Brasília DF 70770-017, Brazil; simone.ribeiro@embrapa.br; 4 Departamento de Biologia Celular, Campus Universitário Darcy Ribeiro, Universidade de Brasília (UnB), Brasília DF 70910-900, Brazil; rresende@unb.br (R.O.R.); aneliseorilio@gmail.com (A.F.O.)

**Keywords:** virome, metagenomics, *Hovenia dulcis*, HDaV1, HDaV2, *Picornavirales*

## Abstract

In a systematic field survey for plant-infecting viruses, leaf tissues were collected from trees showing virus-like symptoms in Brazil. After viral enrichment, total RNA was extracted and sequenced using the MiSeq platform (Illumina). Two nearly full-length picorna-like genomes of 9534 and 8158 nucleotides were found associated with *Hovenia dulcis* (*Rhamnaceae* family). Based upon their genomic information, specific primers were synthetized and used in RT-PCR assays to identify plants hosting the viral sequences. The larger contig was tentatively named as *Hovenia dulcis*-associated virus 1 (HDaV1), and it exhibited low nucleotide and amino acid identities with *Picornavirales* species. The smaller contig was related to insect-associated members of the *Dicistroviridae* family but exhibited a distinct genome organization with three non-overlapping open reading frames (ORFs), and it was tentatively named as *Hovenia dulcis*-associated virus 2 (HDaV2). Phylogenetic analysis using the amino acid sequence of RNA-dependent RNA polymerase (RdRp) revealed that HDaV1 and HDaV2 clustered in distinct groups, and both viruses were tentatively assigned as new members of the order *Picornavirales*. HDaV2 was assigned as a novel species in the *Dicistroviridae* family. The 5′ ends of both viruses are incomplete. In addition, a nucleotide composition analysis (NCA) revealed that HDaV1 and HDaV2 have similarities with invertebrate-infecting viruses, suggesting that the primary host(s) of these novel virus species remains to be discovered.

## 1. Introduction

Natural forest ecosystems and cultivated forest plantations are responsible for covering around 30% of the entire surface of the Earth [1]. The economic exploitation of forests provides a wide range of benefits, including the production of food, timber wood, charcoal, and pharmaceutical and cosmetic products, among other items. Importantly, the conservation and expansion of native forests generate positive impacts in carbon sequestration as well as in preserving fauna and flora diversity and mitigating the deleterious effects of climate change [1].

Tree species are affected by various pathogens, which are responsible for extensive economic and ecological damages [2]. Native and cultivated tree species may serve as alternative sources of inoculum of viruses that can infect other economically important crop species. Therefore, information about the viral diversity associated with forest host species is critical and provides the basis for the establishment of effective disease management and control strategies. Several studies of viruses occurring in the temperate forests of Europe have been carried out [3]. However, the characterization of viruses infecting trees, especially in Neotropical areas, is yet scarce [4]. In Brazil, the pioneering studies on the characterization of viruses on natural forest ecosystems started in the late 1970s [5], allowing the identification of virus species classified in the genera *Carlavirus*, *Orthotospovirus*, *Potyvirus*, and *Tymovirus* [6,7,8,9].

The metagenomic/ecological genomic strategies, coupled with large-scale sequencing platforms, increased the knowledge about the microbial diversity across a wide range of natural environments [10] and has contributed to detect, identify, and characterize several new plant-associated new viruses and viroids without prior knowledge of their genomes [11,12]. A further advantage of the metagenomic strategies is the detection of plant-associated viral sequences, even at low concentrations in their host tissues [13]. A wide range of protocols for enriching virus particles have been used, and several viruses have been detected and characterized after employing these approaches [14,15,16,17,18]. However, the metagenomic characterization of viruses and viroids in tree species is limited to species into the genera of high economic relevance such as *Prunus*, *Pyrus*, *Malus, Citrus*, *Actidinia*, *Diospyros*, *Morus,* and *Vitis* [19,20,21]. Here, we describe the near full-length genomes of two putative novel virus species within *Picornavirales* associated with leaf samples from *Hovenia dulcis* Thumb. (Rhamnaceae family).

## 2. Material and Methods

### 2.1. Plant Material

Leaves of tree seedlings displaying virus-like symptoms were collected at the NOVACAP II (Companhia Urbanizadora da Nova Capital do Brazil) nursery. At the time of collection, the seedlings were about seven months old. A total of 60 plant samples were obtained from 27 native and exotic species from 14 botanical families. Before the enrichment of viral particles, leaf samples were collected from all the seedlings, gently cleaned with a brush, and stored at −80 °C.

### 2.2. Enrichment of Viral Particles

Three individual pools (including 20 samples each, with a total weight of 10 g for each pool) were ground on liquid nitrogen. Afterward, 100 mL of 0.1 M sodium phosphate buffer, pH 8.0 containing 1 mM EDTA, and 0.2% β-mercaptoethanol were added to each sample pool. The samples were macerated, filtered using cheesecloth, and centrifuged (at 2800× *g* for 20 min). The aqueous layer was transferred to ultracentrifuge tubes, and with a long needle, a layer of a sucrose solution (20%) was added into the tube bottom to form a sucrose cushion. Subsequently, the samples were submitted to analytical ultracentrifugation at 4 °C and 33,000× *g* for 2 h. RNA extraction was carried out from the pellet fraction, employing TRIzol reagent (Life Technologies, Carlsbad, CA, USA). Pellets were resuspended in 1 mL of TRIzol and transferred to a fresh tube containing 200 μL of chloroform. All samples were mixed to form a single pool (including all 60 samples) and vigorously vortexed for 15 s and left at room temperature for 3 min. Subsequently, the samples were centrifuged at 12,000× *g* for 15 min at 4 °C. The aqueous layer (1 mL) was transferred to a new tube, and 500 μL of isopropanol was added. The samples were kept at room temperature for 10 min. After that, the samples were again centrifuged (at 12,000× *g* for 10 min at 4 °C). The supernatant was discarded, and 1 mL of 75% ethanol was added to wash the pellet. After centrifuging at 7500× *g* for 5 min, the ethanol was carefully discarded, and the samples left on ice for 2 min. Finally, the RNA was resuspended in RNase-free DEPC (Diethyl pyrocarbonate)-treated water, left on ice for 15 min, aliquoted and stored at −80 °C.

### 2.3. High Throughput Sequencing and Analysis

The pooled RNA was sequenced using the MiSeq sequencing platform (Illumina, San Diego, CA, USA) at the Universidade Católica de Brasília (UCB). Total RNA was converted to cDNA using random hexamers, the library was prepared with Nextera™ DNA Sample Prep Kit and sequenced using MiSeq Reagent Kits v2 (2 × 150 bp) (Illumina, San Diego, CA, USA). The raw reads were quality trimmed and assembled de novo using the CLC Genomics Workbench (v 8.0, Qiagen, CA, USA). The resulting contigs were compared to the complete viral RefSeq database using BLASTx and Blastp algorithms [22] implemented in Geneious program v. 9.1.3 [23]. All sequences with hits matching the viral database were then subjected to a BLASTx search against the complete nr database to exclude false positives. To confirm the assembly results and further extend incomplete genomes, trimmed reads were mapped back to the viral contigs and reassembled until genome completion or no further extension. Genomic regions covered by less than three sequence reads were amplified by RT-PCR (see Table 1 for primer sequences), and Sanger sequenced. The final contigs were annotated using Geneious program (v. 9.1.3, Biomatters, Auckland, New Zealand) [23]. The 3’ends secondary structure were predicted using the RNAfold web server (http://rna.tbi.univie.ac.at//cgi-bin/RNAWebSuite/RNAfold.cgi) [24,25,26]. The sequences were deposited at the GenBank under the accession numbers MT079817 and MT079818.

### 2.4. RNA Extraction and Virus Detection by RT-PCR

Based on the assembled contigs, a set of specific primers were designed to determine the presence or absence of each of the five viruses and used to assay each sample by RT-PCR (Table 1). The total RNA was individually extracted from each of the 60 original samples using the Hot Phenol protocol [27]. All centrifugation steps were carried out at 4 °C. The purity and integrity of the RNA were confirmed by electrophoresis on a 1% agarose gel. Complementary DNA (cDNA) was synthesized with the viral-specific reverse primers. The reaction was performed using the Moloney Murine leukemia Virus Reverse Transcriptase (M-MLV) (Invitrogen, Carlsbad, CA, USA) according to the manufacturer’s instructions. Initially, a mixture of 3.5 µL of RNAse-free water, 4.5 µL of RNA, 1 µL of reverse primer (10 µM), and 1 µL of dNTP (10 mM) was incubated at 70 °C for 5 min. Then 3 µL of M-MLV 5× buffer [250 mM Tris-HCl (pH 8.3); 375 mM KCl; 15 mM MgCl_2_, and 0.1 M DTT], 1 µL of M-MLV enzyme (200 U/µL), 1 µL of RNase OUT (40 U/µL) (Invitrogen, Carlsbad, CA, USA) 1 µL of 100 mM DTT, and 4 µL of RNAse-free water were added to, in a total volume of 20 µL. Samples were incubated at 37 °C for 60 min and 15 min at 70 °C. The PCR assays were performed in a total volume of 12.5 µL. The reaction was composed by 8.0 µL of DNAse-free water, 1.25 µL of buffer 10X, 0.4 µL of MgCl_2_ (50 Mm), 0.25 µL of dNTP (10 mM), 0.25 µL reverse primer (10 µM), 0.25 µL of forward primer (10 µM), 0.1 µL of *Taq* DNA polymerase (500 U/µL) (Invitrogen, Carlsbad, CA, USA), and 2 µL of cDNA. The PCR parameters were as follows: initial denaturation of 94 °C for 2 min followed by 34 cycles of denaturation (94 °C for 30 s), annealing (58 °C for 45 s), and extension (72 °C for 1 min). A final extension step (72 °C for 7 min) was employed. The correct size PCR products were identified by gel electrophoresis (1%), gel-purified, cloned into pGEM-T Easy Vector (Promega, Madison, WI, USA), and Sanger sequenced at CNPH (Centro Nacional de Pesquisa de Hortaliças, Brasília, Brazil).

### 2.5. 3′ RACE

The 3′ end of the genomes was amplified using the 3′ RACE, as described by [28,29]. Briefly, the cDNA was synthesized using an oligo(dT) primer with an anchor sequence (Oligod50TM4) (Table 1) and SuperScript™ III Reverse Transcriptase (Invitrogen, Carlsbad, CA, USA), according to the manufacturer’s instructions. The PCR was performed using this cDNA with virus-specific forward primers (Table 1) and the anchor reverse primer M4. The PCR products were identified by gel electrophoresis (1%), gel-purified, and Sanger sequenced at CNPH.

### 2.6. Phylogenetic Analyses

Phylogenetic analyses were carried out with RNA-dependent-RNA-polymerase (RdRP) protein sequences from members belonging to the order *Picornavirales*. The amino acid sequences were aligned using ClustalW [30] implemented in Geneious [23]. The maximum likelihood tree was inferred with the FastTree algorithm [31] implemented in Geneious, with JTT+CAT [32]. Branch support was estimated using the non-parametric Shimodaira–Hasegawa-like approximate likelihood ratio test (SH-aLRT) [33]. The genome organization of each of the viruses was annotated on the tree using the Evolview v3 server [34]. The accession numbers of sequences used in the alignment are displayed in Table S1.

### 2.7. Nucleotide Composition Analysis (NCA)

The nucleotide composition analysis (NCA) method was used to infer the most likely virus host in this study [35]. For NCA, a dataset of 278 complete genomes sequences with defined host origins (e.g., insects, vertebrates, plants, algae, protozoans, and environmental samples) and also comprising genomes from species, genera, and families within the order *Picornavirales* were retrieved from the NCBI/GenBank (https://www.ncbi.nlm.nih.gov/). In viruses with bipartite genomes, the sequences of the components were concatenated, and these were considered as the complete genome. A Linear Discriminant Analysis (LDA) was performed to identify the most likely host species of the viruses reported in the present work. Dinucleotide frequencies for each sequence were determined using the program simple sequence editor (SSE), version 1.3 [36]. LDA was performed using the R program (www.R-project.org) (version 3.4.2) [37], implementing the MASS, LDA function, and ggplot2 package. The accession numbers and hosts of the genomes employed in the NCA are presented in Table S2.

## 3. Results

**Figure 1:**
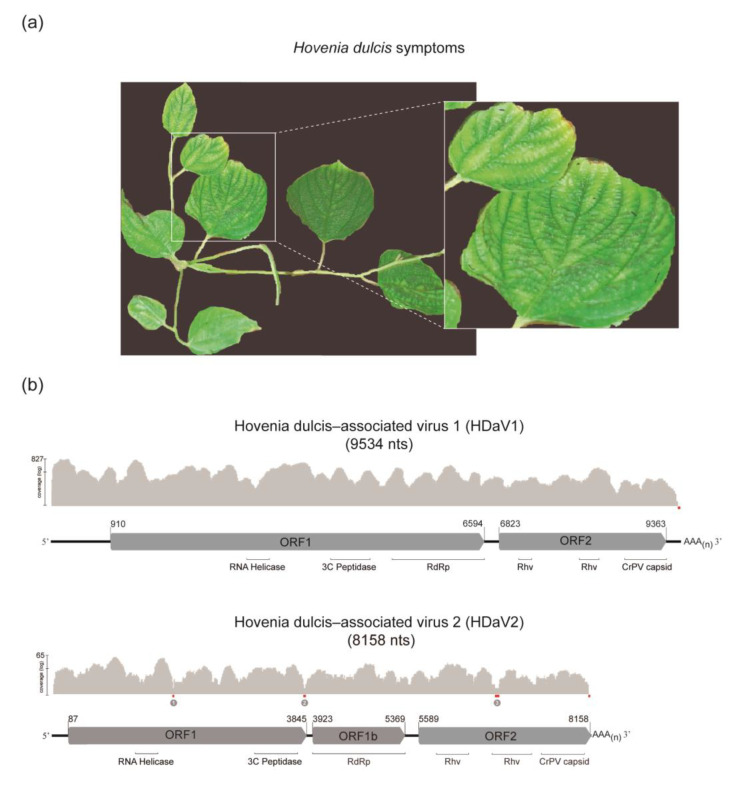
*Hovenia dulcis* symptoms and viral genomic organization. (**a**) Virus-like symptoms observed in leaves of *Hovenia dulcis*: interveinal chlorosis. (**b**) Schematic representation of *Hovenia dulcis*-associated virus 1 (HDaV1) and *Hovenia dulcis*-associated virus 2 (HDaV2) genomic organization and sequencing coverage. All open reading frames (ORFs) are represented as arrows pointing from the 5′ to the 3′ end and are colored in grey. Nucleotide positions indicate the start and end of ORFs. ORF 1 and 1b encodes non-structural polyproteins, including putative functional domains (RNA Helicase; 3C peptidase protease; and RdRp: RNA-dependent RNA polymerase). ORF 2 encodes a structural polyprotein with capsid protein domains (Rhv: picornavirus (Rhinovirus) capsid protein-like, CrPV capsid: cricket paralysis virus capsid protein-like). Regions with low coverage are highlighted in red, and the numbered regions were confirmed by RT-PCR and Sanger sequencing.

As part of a field survey for plant-infecting viruses, samples from a variety of plant species were collected in 2014 at NOVACAP Nursery, located in Brasília-DF, Brazil. This nursery is responsible for the production of tree seedlings for urban reforestation purposes. We processed and sequenced one pool of samples containing viral enriched RNA from 60 plants, including two plants of *H. dulcis* showing virus-like symptoms (interveinal chlorosis) (Figure 1a). After MiSeq sequencing (Illumina), a total of 5,005,110 raw reads were generated. The raw reads were trimmed and de novo assembled using CLC Genomics Workbench v.8.0 (Quiagen program). The resulting 2162 contigs were compared against a viral RefSeq database using BLASTx algorithm [22] and five contigs were initially assigned to the *Picornavirales* order. While two large contigs (9529 and 8126 nucleotides—nts) were related to unclassified members of *Picornavirales* and *Dicistroviridae*, the remaining three contigs with small sizes (1096, 781, and 635 nts) and relatively low coverage (≤50 reads) were related to Secoviridae members. The presence of these putative novel viruses was investigated by RT-PCR and Sanger sequencing in all samples individually. All sixty samples were negative to the *Secoviridae* related contigs; therefore, they were not further investigated. The two large contigs were detected only in one *H. dulcis* leaf sample. After 3′ RACE, five additional nucleotides from 3′-terminal sequence of the larger contig were recovered, resulting in a final contig with 9534 nts plus the poly(A) tail. Moreover, the minor contig was increased in 32 nts, resulting in a final contig with 8158 nts plus the poly(A) tail.

The nearly full-length genome of 9534 nts (assembled from 8669 reads) displayed two non-overlapping ORFs (Figure 1b). The first ORF (spanning from the nucleotide 910 to the nucleotide 6594) encodes the replication proteins (one helicase, a tyrosine-like serine protease, and RNA-dependent RNA polymerase domains) and the second ORF (spanning from the nucleotide 6823 to the nucleotide 9363) codes for a structural protein (CP domain). The two ORFs are separated by a 228 nts intergenic region (IGR). However, characteristic IRES-like structures were not identified. The 5′-UTR and 3′-UTR ends contain 909 and 171 nts, respectively. Although it is likely that the 5′-UTR is still incomplete, this is one of the largest 5′UTR (909 nts) when compared to other genetically related viruses, while two insect-infecting viruses, Hubei picorna-like virus and Hubei picorna-like virus 79 each have reported 5′-UTR of only 211 nts, suggesting that the 5′-UTR size varies significantly among the order *Picornavirales*.

Pairwise identity comparisons of this contig sequence with those of representative *Picornavirales* members indicated that it shares the highest degree of nucleotide identity (71%) with the Darwin bee virus 6 isolate NT-8 (9123 nts) (MG995696), a yet unclassified *Picornavirales* member reported infecting honey bees (*Apis mellifera*) [38]. Additionally, the putative proteins, encoded by ORF 1 and ORF 2, share 65.5% and 70% aa identity with Darwin bee virus polyproteins (AWK77846 and AWK77847), supporting its classification as a new species according to the species demarcation criteria proposed by the International Committee on Taxonomy of Viruses (ICTV) (i.e., protein identity of less than 90% with its closest relatives) [39]. The name *Hovenia dulcis*-associated virus 1 (HDaV1) is proposed for this virus.

Interestingly, the contig of 8158 nts (assembled from 689 reads) presented three non-overlapping ORFs of 3758 nts (ORF1), 1446 (ORF1b) and 2571 nts (ORF2) (Figure 1b). Since low coverage contigs are more susceptible to sequencing errors and spurious assembly, three pairs of primers were designed to confirm regions with insufficient coverage (Figure 1b, highlighted in red). The sequence generated by Sanger sequencing was identical to the sequence obtained by HTS, except for one Illumina read with an insertion in a T homopolymer (position 3827 to 3830), which would reconstitute the longer ORF1 frame typical of members of the family *Dicistroviridae*. To further investigate this result, we performed a new HTS using total RNA from *H. dulcis* leaves, and no insertion was observed in the reads mapped in this genomic region. However, only a limited number of reads (226 reads) mapped to the HDaV2 genome.

The first two ORFs were separated by a short IGR of 78 nts, and they encode the non-structural protein precursors. The ORF1 putative protein presented the RNA_helicase and 3C peptidase protease domains, whereas ORF1b putative protein presented the RNA-dependent RNA polymerase domain. ORF2 is separated from ORF1b by an IGR of 219 nts and encodes a structural polyprotein, which contains the three capsid domains (Figure 1b). All ORFs are predicted to initiate translation at canonical AUG codons. Moreover, the 5′-UTR and IGR motifs, typical of dicistrovirus [40], were not observed. Unfortunately, some nucleotides at the 5′ end of the genome are missing compared with other related viruses (described below). Interestingly, the poly(A) tail was located immediately downstream of the stop codon, which is an uncommon feature among members of the order *Picornavirales*. A hairpin structure was predicted at nucleotide positions 8124–8158 (Figure S1). However, its functionality remains to be evaluated.

The pairwise identity comparisons indicated that it shares the highest degree of nucleotide identity with two viruses reported in *Bemisia tabaci* samples from Brazil: Bemisia associated dicistrovirus 2 (MN231041, unpublished) (BaDV-2) and Bemisia associated dicistrovirus 1 (BaDV-1) (MH459180) [41]. Crucially, BaDV-2 presented the same unusual genomic organization (three non-overlapping ORFs) observed above. A comparison of these genomes revealed a single nucleotide deletion that produces two ORFs (ORF1 and ORF1b), which do not occur in BaDV-1 ORF1 (Figure S2). Based on BLASTp analysis, BaDV-2 shared 48%, 73%, and 69% of an amino acid identity, the putative proteins, encoded by ORF1, ORF1b, and ORF 2, respectively. Moreover, the BaDV-1 polyproteins (AZB50980 and AZB50981) shared 44% and 69% aa identity with ORF1/ORF1b and ORF2, respectively. Overall, these results confirm that HDaV2 represents a new species within the family *Dicistroviridae*, which we have tentatively named *Hovenia dulcis*-associated virus 2 (HDaV2).

**Figure 2:**
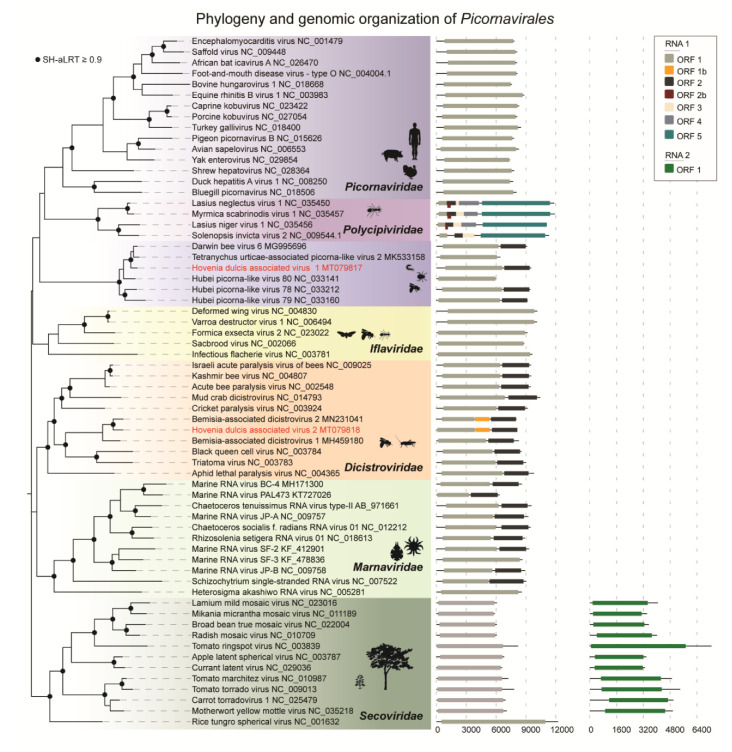
Phylogeny and genomic organization of representative members of the order *Picornavirales*. Phylogenetic analysis based on the amino acid sequence of RdRp (RNA-dependent RNA polymerase) of members from six families within the order *Picornavirales*. Sequences were aligned with ClustalW [30] and the maximum likelihood tree inferred with FastTree [31]. The black circles represent nodes with aLRT ≥0.9. *Hovenia dulcis*-associated virus 1 (HDaV1) and *Hovenia dulcis*-associated virus 2 (HDaV2) are highlighted in red. The genome organization was plotted with the Evolview v3 program [34].

The phylogenetic analysis based on the conserved RdRp domain of the two novel viruses (HDaV1 and HDaV2) and representative viruses in the order *Picornavirales* confirmed the BLASTx and pairwise identity results (Figure 2). HDaV1 showed close relationships to a several unclassified bicistronic picorna-like arthropod-infecting viruses: Darwin bee virus 6 [38], Hubei picorna-like-79, Hubei picorna-like-78, Hubei picorna-like-80 [42], and Tetranychus urticae-associated picorna-like virus 2 [43], forming a potential new family within the *Picornavirales* order (Figure 2). The genomes of Hubei picorna-like virus 80 and Tetranychus urticae-associated picorna-like virus 2 are probably incomplete given the absence of an ORF encoding the structural polyproteins (ORF2). Additionally, HDaV2 clustered with BaDV-1 and BaDV-2 in a monophyletic clade, supporting the notion that these viruses represent new species within a new genus in the family *Dicistroviridae* [41].

**Figure 3:**
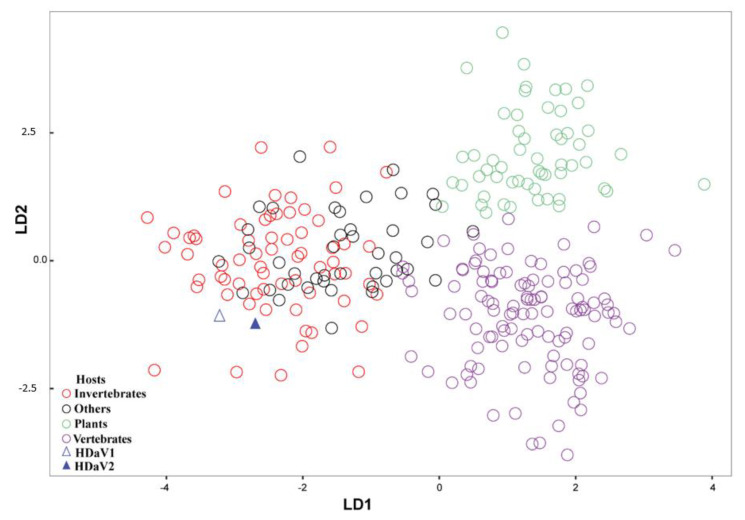
Linear discriminant analysis (LDA) used to classify viral sequences into host groups. Linear discriminant analysis comparing nucleotide composition from members of order *Picornavirales* with known hosts. Invertebrates-infecting viruses are shown in open red circles, plant-infecting viruses are in open green circles, and vertebrate-infecting viruses in open purple circles; *Hovenia dulcis*-associated virus 1 (HDaV1) is indicated by an open blue triangle, and *Hovenia dulcis*-associated virus 2 (HDaV2) is indicated by a solid blue triangle. Other picornaviruses (algae, protozoa, and environmental samples) are shown in open black circles.

A nucleotide composition analysis (NCA) was performed in an attempt to identify the most likely host(s) of the HDaV1 and HDaV2. A total of 278 sequences from the order *Picornavirales* were used, and four pre-defined categories of hosts were used for NCA (viz. invertebrates, others (algae, protozoa, and environmental samples), plants, and vertebrates). As shown in Figure 3, three groups were formed after linear discriminant analysis: plant-infecting viruses (in green), vertebrate-infecting viruses (in purple), and a third mixed group formed by invertebrate- and protist-infecting viruses (red and black). HDaV1 and HDaV2 clustered with invertebrate-infecting viruses (Figure 3).

## 4. Discussion

In a systematic field survey for plant-infecting viruses, leaf tissues were collected from trees showing virus-like symptoms in Brazil. Two putative new ssRNA+ viruses were found in leaves of *H. dulcis* seedlings grown under nursery conditions, confirming that the viral enrichment protocol followed by HTS is a sensible and economic strategy for discovering new viruses, even with the dilution effect of sample-pooling. Based upon the genomic organization and phylogenetic analyzes, these two viruses were tentatively classified as novel viral species within the order *Picornavirales*. We proposed the names *Hovenia dulcis*-associated virus 1 (HDaV1) and *Hovenia dulcis*-associated virus 2 (HDaV2).

The order *Picornavirales* harbors viruses with ssRNA+ genomes, spherical particles with a diameter around 30 nm, distinct genomic organization and segmentation (mono or bipartite), as well as distinct host organisms (algae, insects, protists, plants, and vertebrates) [44]. Currently, the combined analyses of these features allow for the allocation of viral species into six families (*Dicistroviridae*, *Iflaviridae*, *Marnaviridae*, *Secoviridae, Picornaviridae,* and *Polycipiviridae*) [45,46]. HDaV1 and HDaV2 shared many of the key characteristics of the *Picornavirales* members, including the genomic organization with conserved regions with HEL/PRO/RdRp motifs [47]. However, HDaV2 presented a novel genome organization within the family *Dicistroviridae*, with three non-overlapping ORFs.

Importantly, HDaV1 and HDaV2 clustered with invertebrate-infecting viruses, suggesting that they might be (i) invertebrate-infecting viruses derived from some undetected invertebrate that was contaminating our plant samples, (ii) they are bona fide yet unknown plant-infecting viruses, or (iii) they can infect both invertebrates and plants. The presumed relationships with insect-infecting viruses were supported by the NCA results, which grouped HDaV1 and HDaV2 with picornaviruses infecting invertebrate and “other” hosts rather than those infecting either vertebrates or plants (Figure 3). Therefore, an invertebrate or invertebrates are the most likely primary hosts of both HDaV1 and HDaV2. However, it is important to highlight that during sample collection, it was not possible to determine the conspicuous presence of insects or mites. Besides, before viral enrichment process, leaf tissues were carefully cleaned with a brush under a stereo-microscope. Significantly, no reads/contigs were related to *Bemisia tabaci* genes, which was the only insect observed during our surveys. Moreover, dual tropism (invertebrates/plants) have already been described in both invertebrate-infecting viruses or plant-infecting viruses [46,47,48,49,50]. Dual tropism could explain, for example, the presence of virus-like symptoms in the *H. dulcis* leaf samples. For instance, *Rhopalosiphum padi virus*—RhPV (genus *Cripavirus*; family *Dicistroviridae*) is commonly reported infecting aphids, which are well-characterized plant-pests. In this context, plants might also serve as secondary hosts or reservoirs for RhPV, contributing to its horizontal transmission in aphids [48,50,51,52,53]. In some cases, virus replication in both plants and insects has also been confirmed. Tobacco ringspot virus (genus *Nepovirus*, family *Secoviridae*) is a main example, which was found causing systemic infection in *Apis mellifera* [46,47,48]. Recently, the ability of an insect-infecting RNA virus from Lepidoptera to establish infection in cowpea (*Vigna unguiculata* (L.) Walp) as well as in mammalian cell culture lines has been demonstrated, providing evidence of a virus that can infect hosts of distinct kingdoms [54]. We also found three contigs related to family *Secoviridae*, but they were not detected in any plant within the pool, probably due to their relatively low coverage or due to index hopping, which may result in the assignment of sequencing reads to the wrong index during demultiplexing [54].

Therefore, the virus detection exclusively in leaf samples of *H. dulcis* allowed us to speculate that the two new viruses described here could have a close relationship with this plant, even though, no viral movement protein was identified in the HDaV1 and HDaV2 genomes. In addition, no other plant sampled in the same area was found to be positive for either HDaV1 or HDaV2, reinforcing the hypothesis that these viruses might be exclusively associated with either *H. dulcis* or with some yet unidentified arthropod pest of this plant species. In this context, further biological assays should be performed to elucidate the interaction among *H. dulcis* and both viruses.

## Figures and Tables

**Table 1:** Specific primers sequences used in PCR, RT-PCRs, and 3′ RACE.

Primer Name	Primer Sequence 5′–3′	AT ^1^ (°C)	Amplicon Size (bp)	Application/Target Genomic Regions
HDaV1_7626_F ^2^	AGTCACTGGTGCGTTAGGTG	57	993	Detection/Capsid
HDaV1_8618_R ^3^	GTAAGCATACCTCCACGCGA			
HDaV2_6983_F	GAATGAACTGCGTGCTACAC	59	757	Detection/Capsid
HDaV2_7739_R	CCGGGGGAAAACAGCAGT			
C1622_254_F ^4^	TTAATGGGGTTGCAGGGCTT	60	519	Detection/RdRp
C1622_772_R ^4^	TCATGACTCCTATGCGCCAC			
C1177_207_F ^5^	GTGTCGTTTGTATCGCAGGC	59	674	Detection/RdRp
C1177_880_R ^5^	CGCGCTCATAGCCAAACAAA			
C_1797_31_F ^6^	ATTGAAAACGCGACCTGCAC	59	571	Detection/RdRp
C1797_601_R ^6^	GCGGGATAAGCTCACCAAGT			
HDaV2_1630_F	TGCAAGAGTACCAGGAACAGAATAAT	54	608	Low coverage region 1/ORF1
HDaV2_2236_R	GCAAGGCCATGATACATGACCA			
HDaV2_3431_F	AGAAAGTGTTTACTATGTAGCACCAACT	59	549	Low coverage region 2/ORF1b
HDaV2_3981_R	CTATTCCTTGGCAGGCTTGACG			
HDaV2_6422_F	GTCTGCTCCTGATGCTAATCCG	58	540	Low coverage region 3
HDaV2_6961_R	GCTGGGACATCATCAAGGGAAC			
Oligod50TM4	GTTTTCCCAGTCACGACTTAATTAA(T)50	65	−	Race cDNA
M4	GTTTTCCCAGTCACGACT	56	−	Race 3′ PCR
HDaV1_9041_F	CCTCAGAAGTTTTCGAGACTGC	56	−	Race 3′ PCR
HDaV2_7256_F	ACCTCACAAATATACTGTTGGTGAGG	60	−	Race 3′ PCR
HDaV2_7518_F	CCTGAACTTGGTATATTGGATGTTCCC	60	−	Race 3′ PCR

^1^ Annealing temperature; ^2^ F: Forward; ^3^ R: Reverse; ^4^ contig1622: *Fabavirus*; ^5^ contig1177: *Fabavirus*; ^6^ contig1797: *Comovirus*.

## Supplementary Materials

Supplementary materials can be found at https://www.mdpi.com/1999-4915/12/9/950/s1. Figure S1: The 3′ ends of HDaV1 and HDaV2 genomes. Figure S2: Alignment of a selected region of HDaV2 and two related viruses (BaDV-1 and BaDV-2). Table S1: The accession numbers of the sequences used in the phylogeny. Table S2: The accession numbers of the virus sequences and their hosts used in the nucleotide composition analysis (NCA).
