# Supplementary material for: Molecular Characterization of Hovenia Dulcis-Associated Virus 1 (HDaV1) and 2 (HDaV2): New Tentative Species within the Order Picornavirales
Source: Viruses. 2020 Aug 27;12(9):950. doi: 10.3390/v12090950 (PMC7552035; doi:10.3390/v12090950)
Supplement: Supplementary file 1 [file viruses-12-00950-s001.zip › SupplementaryFiles/SupplementaryFigures.pdf]

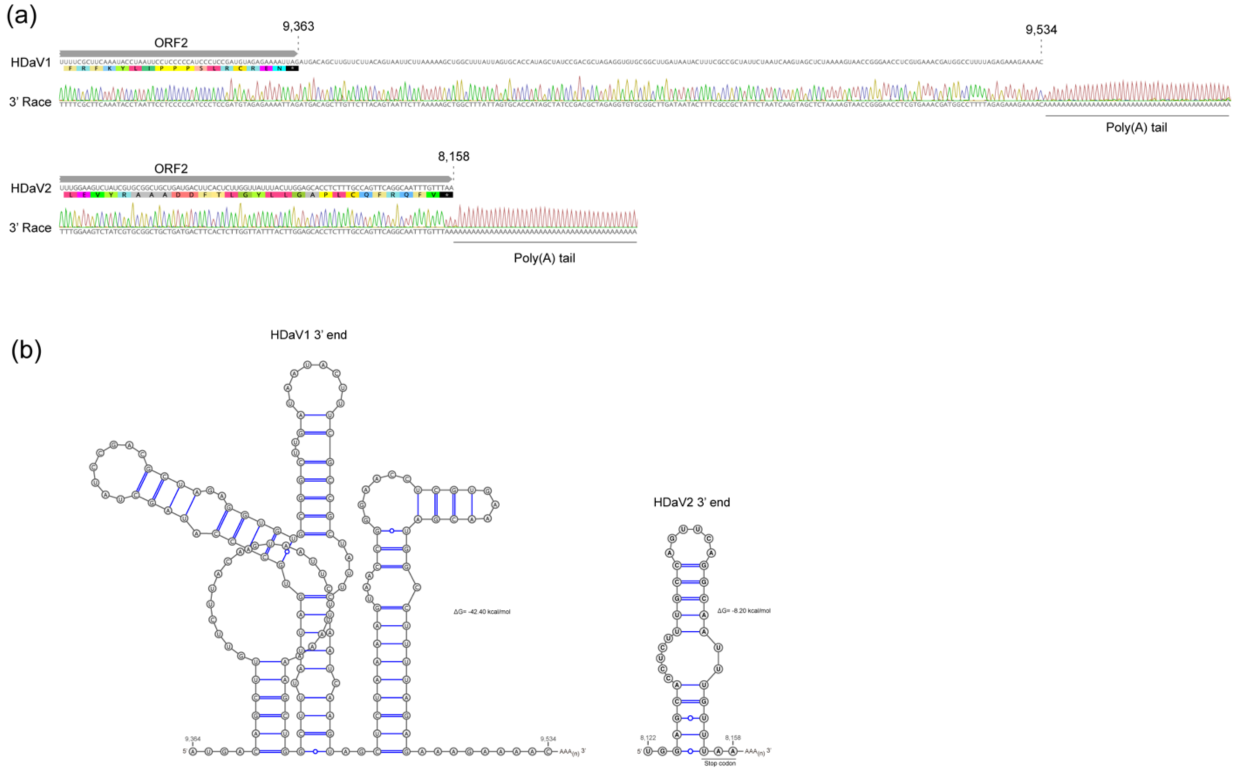

Supplementary Figure 1. The 3′ ends of HDaV1 and HDaV2 genomes. (a) Sanger sequencing of the 3′ RACE products. The ORF2 is presented as a grey arrow. (b) The optimal secondary structure predicted for the 3′ends of HDaV1 and HDaV2 using the RNAfold web server (http://rna.tbi.univie.ac.at//cgi-bin/RNAWebSuite/RNAfold.cgi).

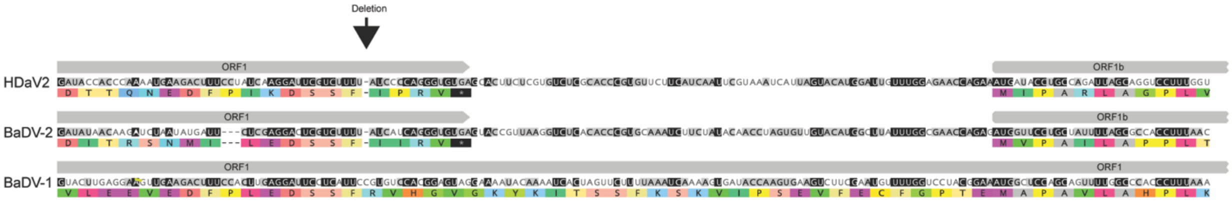

Supplementary Figure 2. Alignment of a selected region of HDaV2 and two related viruses (BaDV-1 and BaDV-2). All ORFs are represented as arrows pointing from the 5′ to the 3′ end and are colored in grey. The protein translation is below the nucleotide sequence. The arrow shows a single nucleotide deletion that generates the two ORFs (ORF1 and ORF1b) in HDaV2 and BaDV-2.
